# Clinical Prognosis for SAH Consistent with Redox Imbalance and Lipid Peroxidation

**DOI:** 10.3390/molecules25081921

**Published:** 2020-04-21

**Authors:** Iwona Jarocka-Karpowicz, Anna Syta-Krzyżanowska, Jan Kochanowicz, Zenon Dionizy Mariak

**Affiliations:** 1 Department of Analytical Chemistry, Medical University of Bialystok, Mickiewicza 2D, 15-222 Bialystok, Poland; 2 Stroke Unit, J. Sniadecki Provincial Hospital of Bialystok, M. Sklodowskiej-Curie 25, 15-950 Bialystok, Poland; annasyta@op.pl; 3 Department of Neurology, Medical University of Bialystok, M. Sklodowskiej-Curie 24A, 15-276 Bialystok, Poland; neurosek@umb.edu.pl; 4 Department of Neurosurgery, Medical University of Bialystok, M. Sklodowskiej-Curie 24A, 15-276 Bialystok, Poland; nch@umb.edu.pl

**Keywords:** lipid peroxidation, antioxidants, subarachnoid hemorrhage, aneurysm

## Abstract

Subarachnoid hemorrhage (SAH) accounts for 3% of all strokes. As more and more data indicates the role of oxidative stress in acute brain damage caused by SAH, an attempt was made to correlate the clinical status of patients with systemic level of antioxidants and lipid peroxidation products. The hemorrhage was diagnosed with brain computed tomography (CT) and aneurysm with angio-CT and angiography, while the vasospasm was monitored with transcranial Doppler. Plasma glutathione peroxidase activity (GSH-Px) and vitamin A, E, and C levels were determined spectrophotometrically and by HPLC, respectively. The levels of polyunsaturated fatty acids (PUFAs) cyclization products were determined by GC–MS, while F_2_-isoprostanes and neuroprostanes (NP) were determined by LC–MS. SAH was accompanied by changes in antioxidant capacity in blood plasma, including initially (day 1) an increase in GSH-Px activity, followed by its decrease and a progressive decrease in glutathione (GSH) levels and vitamins A, E, and C. On the other hand, levels of PUFAs cyclization products, F_2_-isoprostanes, and neuroprostanes were highest on day 1 (two and eight times higher, respectively) and decreased over time. The levels of 4-HNE (4-hydroxynonenal), 4-ONE (4-oxononenal), and MDA (malondialdehyde) changed similarly. In contrast, the 4-HHE (4-hydroxyhexenal) level reduced after SAH increased significantly after a week. It was found that the deterioration of the overall clinical and neurological condition of SAH patients due to cerebral edema, intracranial hemorrhage, or vasoconstriction corresponded to reduced antioxidant defense and, as a consequence, increased lipid peroxidation and slower observed changes in regression. It can be concluded that monitoring the level of lipid peroxidation products (neuroprostanes, 4-ONE, and MDA) can support the monitoring of the clinical status of patients, especially with regard to the assessment of vasospasm.

## 1. Introduction

Intracranial aneurysms are a common problem that affects about 1–2% of the population. They may remain stable over time, but in some cases the aneurysm wall dies and the matrix degenerates, eventually causing a wall rupture with life-threatening hemorrhage [1]. Spontaneous rupture of the brain aneurysm leads to subarachnoid hemorrhage (SAH), a disease that is characterized by significant morbidity and mortality (10–11/100,000 people around the world). Due to the younger age of onset and high mortality compared to other cardiovascular diseases, SAH requires immediate diagnosis and treatment [2]. It is particularly important to prevent systemic and cerebral complications that significantly worsen the prognosis and are associated with the appearance of neurological deficits leading to patient disability [3]. 

Increasing evidence indicates the contribution of oxidative stress to the mechanism of acute brain damage causing cerebral vasoconstriction resulting from SAH [4]. Under physiological conditions, redox homeostasis is the result of a balance between the production of reactive oxygen species (ROS) and the action of antioxidant mechanisms. Because even under physiological conditions, the brain has high pro-oxidative potential and high metabolic requirements at the same time, brain is particularly susceptible to oxidative stress [5,6]. The main source of ROS, in the brain, is the leakage of superoxide anions from mitochondria due to an ischemic disruption of the electron transfer chain and the cascade of free radicals produced from the auto-oxidation of hemoglobin, as well as enzymatic reactions [7]. The cascade of ROS produced during the auto-oxidation of hemoglobin includes mainly superoxide anions and hydrogen peroxide [8]. The concentration of non-bound iron ions in heme is high in several regions of the brain. These ions, as well as ions released from oxyhemoglobin, may catalyze the generation of damaging hydrogen peroxide and hydroxyl radicals, which may also be generated from superoxide anions by the cascade of radical reactions [4,9]. Moreover, several activated pro-oxidant enzymes, phospholipases, NADPH and xanthine oxidases, and nitric oxide synthase, generate superoxide anions [10,11]. Metabolic changes observed in the brain after SAH are associated among others with an increased production of ROS. The rupture of an aneurysm results in hemolysis of erythrocytes, which results in the release of their cytosolic compounds into the subarachnoid space which consequently leads to enhanced ROS generation. Additionally, a pro-inflammatory response in the brain may activate glial cells, resulting in ROS generation [12]. The damaging effects of ROS are limited by numerous cellular antioxidant defense mechanisms; however, the brain antioxidant defense is rather modest [13]. Under physiological conditions, antioxidant enzymes metabolize ROS, but excessive production of ROS resulting from SAH after ischemia may prevent their effective elimination [14]. In addition, SAH may contribute to a decrease in the effectiveness of antioxidant enzymes due to a decrease in the availability of copper and zinc ions and, consequently, a reduction in the activity of superoxide dismutase (Cu, Zn-SOD) as shown in a rat model of SAH or overexpression of this enzyme in humans [15,16]. Under physiological conditions, superoxide dismutase is responsible for superoxide dismutation, producing hydrogen peroxide, which is removed by glutathione peroxidase, which prevents the formation of hydroxyl radicals. However, the increase in superoxide dismutase activity relative to glutathione peroxidase in the case of SAH leads to an increase in the level of hydroxyl radicals and increased oxidative processes and modification of cell components, which leads to a disturbance of metabolic pathways and brain signaling [5].

In connection with the above purpose of this research impact assessment ruptures of intracranial aneurysm on systemic redox balance and lipid metabolism within the first week after aneurysm rupture. An important element of this study is the correlation of changes in biochemical parameters of blood plasma with the clinical condition of patients aimed at indicating an additional diagnostic parameter, which will be also a prognostic indicator of improving the patient’s condition.

## 2. Results

**Figure 1:**
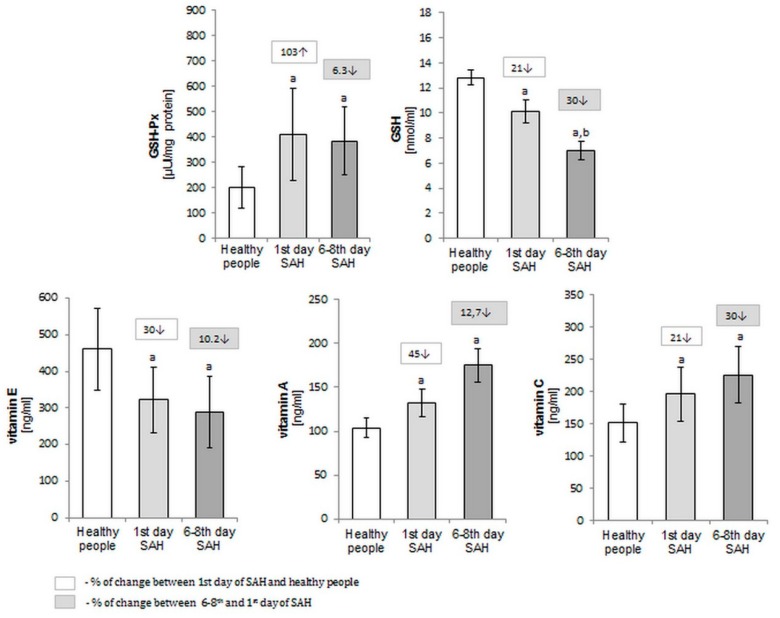
The antioxidants activity (GSH-Px) and level (GSH and vitamins A, E, and C) in the plasma of healthy people (*n* = 30) and SAH-patients (*n* = 30) at the 1st and 6–8th day after SAH; ^a^ *p* < 0.05 in comparison with healthy people. ^b^ *p* < 0.05 in comparison with patients on the 1st day after SAH.

**Figure 2:**
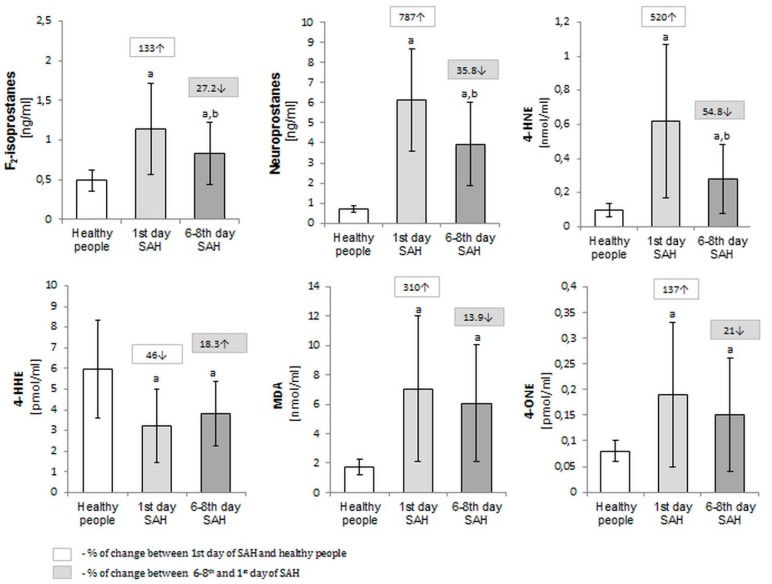
The lipid peroxidation products (F_2_-isoprostanes, neuroprostanes, 4-HNE, 4-HHE, MDA and 4-ONE) level in plasma of healthy people (*n* = 30) and SAH- patients (*n* = 30) at the 1st and 6–8th day after SAH.; ^a^ *p* < 0.05 in comparison with healthy people. ^b^ *p* < 0.05 in comparison with patients on the 1st day after SAH.

The values of all components of the lipid profile and antioxidants show a significant difference between SAH patients and the corresponding matched controls. Plasma levels of antioxidant and lipid peroxidation products were measured in healthy subjects and SAH patients during admission to the hospital and in the 6 to 8 days after hospitalization, and are listed in Figure 1 and Figure 2, respectively. Even considering the protective systems against free radical production, we observe elevated levels of GSH-Px during hospitalization, and a decreased concentration of vitamin A, E, and C (Figure 1).

SAH leads to significant, iterative increase in plasma level lipid peroxidation products such as F_2_-isoprostanes, neuroprostanes, 4-HNE, MDA, and 4-ONE in comparison to healthy subjects. One exception is the concentration of 4-HHE, where a successive decrease was observed from the date of onset, with a mean concentration of 3.23 ± 1.78 pmol/mL to 3.82 ± 1.56 after 6 to 8 days of hospitalization. The mean value of F_2_-isoprostanes and neuroprostanes concentrations were increased 133% and 787% respectively in SAH-patients in comparison with the control group (more than 2-fold for F_2_-isoprostanes, and 9-fold for neuroprostanes) (Figure 2).

After taking a medical history, each SAH-patient was examined neurologically during admission to the hospital. Neck stiffness was observed in all patients, while Kernig’s sign was present in 64% of patients. The following focal symptoms were observed during SAH: hemiparesis, blurred vision, and speech impairment in the form of motor and mixed aphasia. These symptoms occurred in 5 patients, while the remaining patients presented only neck stiffness. The Hunt and Hess scale was used to classify the patient’s neurological condition along with CT scans.

The dependence of antioxidants and lipid peroxidation product concentration was assessed in relation to the World Federation of Neurological Surgeons Scale. The clinical grading system is based on Glasgow Coma Scale (GCS), and the presence of neurological symptoms. We also compared the first degree without motor deficits, observed in 23 people, with patients in the other stages. We observed 4 patients in the second degree with impaired orientation and 3 patients in the fourth degree with motor deficit and significant impairment of consciousness. The results presented in Table 1 and Table 2 show that worsened neurological condition in poor grade patients comes with a higher increase in the concentration of 4-HNE, F_2_-isoprostanes, MDA, and neuroprostanes.

The Fisher scale is based on changes observed in the brain CT performed for each patient during admission to hospital. First and second degrees were observed in 11 patients with a small amount of blood in the subarachnoid space. In stage III, 4 patients presented with hematoma in the subarachnoid space, and 15 patients with blood perforation into the ventricular system. Intracerebral hematoma was observed in 6 patients. It has been shown that blood volume impacts the growth of F_2_-isoprostanes, 4-HNE, 4-ONE, and MDA concentration and the greater decline in vitamin A and E and 4-HHE levels.

Edema in the brain CT was observed in 19 patients with SAH. Analysis of oxidative stress molecules demonstrated increased levels of neuroprostanes, MDA, and 4-ONE. During brain edema, increased GSH-Px and a greater reduction of vitamins A and E were observed.

Each patient hospitalized in the course of SAH was monitored with transcranial Doppler (TCD) for the vasospasm. This complication occurred in 16% of patients. Evaluated antioxidant and lipid peroxidation products levels are shown in Table 1 and Table 2 respectively. Patients presenting with vasospasm had increased average values of MDA and 4-ONE and a decreased decline of neuroprostanes and 4-HNE. Analyzing the concentration of vitamin C, a gradual increase 6 to 8 days after SAH was observed in patients with a better clinical condition. In contrast, a continued decline in the same period was detected in patients with complications. The treatment performed for SAH-patients was embolization in 19 cases. In the remaining 11 patients, after angiography, aneurysm clipping was performed. The above study recorded only one death. As the results show, changes in biochemical parameters are partly dependent on the sex of patients. Men were characterized by a greater reduction in vitamins A and E compared to women after a week of hospitalization. Significant changes depending on the sex of patients were also observed in the level of lipid peroxidation products. In the period 6 to 8 days after SAH, the levels of neuroprostanes and 4-HNE in women’s plasma decreased more strongly, and the levels of F_2_-isoprostanes decreased more significantly in men’s plasma.

## 3. Discussion

Intracranial aneurysm, usually arising in the bifurcation of the cerebral artery, is the result of pathological changes that reduce the tensile strength of the arterial wall and hemodynamic blood flow to which endothelial cells are exposed [17]. It is suggested that mast cells contribute to atherosclerosis and aneurysm formation, which can exacerbate CNS (central nervous system) damage in ischemic and hemorrhagic stroke models by enhancing inflammatory responses and promoting brain–blood barrier disruption, cerebral edema, extravasation, and SAH [18,19]. Moreover, an increase in the level of macrophages, T-lymphocytes, and leukocytes in the wall leads to increased production of ROS, which can modify proteins and lipids, including LDLs (low density lipoproteins), causing atherosclerotic changes in the walls of the vessels. This can lead to extracellular matrix degradation and an increased risk of arterial wall rupture [20,21,22]. The loss of endothelium continuity results in subarachnoid hemorrhage and exposure of collagen, which leads to thrombus formation in which blood cells become trapped. However, the subarachnoid hemorrhage results in release of pro-inflammatory cytokines such as IL-1, IL-6, and TNF-α from microglia and macrophages, resulting in local and systemic inflammation [23,24]. In addition, several brain structures, such as black matter and the nuclear shell, are predisposed to high ROS production due to a high content of iron ions, which can catalyze the formation of hydroxyl radicals. Increased ROS generation was also observed in ischemic and hemorrhagic stroke, with oxidative stress being one of the causative mechanisms of tissue damage in these diseases [25].

Under physiological conditions, ROS are balanced by antioxidant mechanisms, including enzymatic and non-enzymatic antioxidants. Blood extravasation into the subarachnoid space as a result of aneurysm rupture leads to a decrease of cerebrospinal fluid superoxide dismutase activity and reduced antioxidant capacity [26]. Animal studies have shown that after SAH, the activity of superoxide dismutase, which catalyzes the dismutation of superoxide anions to hydrogen peroxide, is increased, and iron ions released from hemoglobin participate in the Fenton reaction, expanding the cascade of radical reactions [16]. However, it should be remembered that these data come from preclinical studies whose reference to people would require standardization. The activity of glutathione peroxidase, which removes peroxides including lipid peroxide, is also increased after SAH [27], which is confirmed by the results presented in this study. In addition, the consequence of SAH is also a reduced level of GSH, which is a GSH-Px co-substrate responsible for regulating glutathione peroxidase activity. Consequently, the efficiency of lipid peroxide removal by glutathione peroxidase is limited. This is especially important because it relates to the critical need to protect brain phospholipids from peroxidation. In addition, the non-enzymatic lipophilic antioxidant, vitamin E, which protects lipids from peroxidation, is not very effective due to the reduced level after SAH. The reduced efficiency of vitamin E is also affected by the reduced level of glutathione and vitamin C [28]. In patients with focal symptoms and in a generally more severe neurological conditions associated with the presence of intracerebral hematoma and cerebral edema, a much more pronounced decrease in vitamin E levels was observed. In the context of obtained data, positive results of vitamin E therapy used in patients with ischemic stroke can be explained [29,30]. Similarly as in the case of vitamin E, it was found that the levels of another lipophilic antioxidant, vitamin A, an antioxidant that cooperates with vitamin E and vitamin C, is reduced as a result of metabolic changes caused by SAH and remains at a reduced level for another week.

Brain cells, especially nerve cell membranes, are rich in phospholipids that contain high levels of PUFAs, in particular arachidonic, linoleic, and docosahexaenoic acids, which due to the content of unsaturated bonds are extremely susceptible to ROS with the generation of reactive aldehydes, which are products of oxidative fragmentation and derivatives of prostaglandins—the products of oxidative cyclization of hydrocarbon chains of acids [31,32,33]. Due to the presence of DHA in neurons and its greater susceptibility to oxidation than AA (arachidonic acid), neuroprostanes are formed, which are useful for assessing brain disorders, especially when identified by assessing systemic changes [34]. Neuroprostanes are present in the plasma of healthy individuals but are dramatically elevated, more than eight-fold, in the plasma of SAH patients. Such an increase may also suggest that the inflammatory response and oxidative stress caused by SAH are not only limited to the brain. It was found that the level of neuroprostanes decreases gradually and statistically significantly during recovery, especially in the case of patients with a better neurological condition. However, in the case of major bleeds, with volume estimated by CT, a significantly elevated level of neuroprostanes was found, which is confirmed by literature data [35]. Our results also show an increase in the level of the product of DHA oxidative fragmentation—4-HHE immediately after SAH and in the next days, especially in the case of severe hemorrhage, edema, hematomas, and intracerebral complications such as vasospasm. This DHA response to SAH indicates a greater DHA preference for oxidative fragmentation than cyclization under SAH conditions [36]. However, during oxidative cyclization of AA F_2_-isoprostanes are primarily generated. These prostaglandin derivatives are similar to neuroprostanes and are relatively unreactive compounds, considered to be the most reliable biomarkers of oxidative stress [37]. Compared to the control, F_2_-isoprostanes levels were more than two-fold higher immediately after SAH, with even higher values for patients with neurological deficits related in particular to the presence of a cerebral hematoma.

Notwithstanding the oxidative fragmentation of DHA, AA and LA (linoleic acid), also undergo metabolism with the formation of 4-HNE and 4-ONE, whose levels were the highest one day after SAH, and then a smaller decrease was observed in patients with hematoma and complications in the form of vasospasm along with a larger increase in 4-HNE concentration immediately after SAH. However, it is known that 4-HNE is toxic to neurons and interferes with glutamate uptake, as well as mitochondrial function at synapses, leading to excitotoxic neuronal death [38]. A similar direction of changes was observed in the case of level of MDA—n-3 and n-6 were the PUFAs peroxidation products [39]. Patients with severe neurological conditions, with complications and additional negative changes determined by brain CT, had a higher MDA concentration eight days post-SAH compared to the values observed in patients of better neurological conditions. Thus, MDA concentration strongly correlated with level of improvement during treatment of SAH. The increase in serum MDA levels in patients with brain disorders has previously been reported [40]. All the examined aldehydes are highly reactive and can act as “secondary messengers” to primary free radicals through reactions with nucleophilic groups of proteins, lipids, and DNA and the formation of stable adducts [41]. Therefore, not only aldehydes but also their adducts are increasingly indicated as diseases biomarkers, including CNS diseases [42].

To relate the changes in biochemical parameters associated with oxidative stress observed after SAH with damage to brain structures, CT results were used as reference. Therefore, it should be considered that the administered contrast compound may also affect plasma redox balance, as it is known that contrast may affect various blood biochemical parameters [43]. However, with the fact that the elimination half-life of the compound used as a contrast agent in this study is about 2 h [44], while the analysis of the parameters assessed in this study took place about 20 h after contrast administration, it could be assumed that the contrast should not disturb the redox balance after this amount of time from exposure.

Neurosurgical treatment of SAH-patients diagnosed with a ruptured aneurysm consists of clipping or coiling as a less invasive technique. Irritation of the arteries is particularly hazardous, which can occur through products generated by the breakdown of hemoglobin. Such irritation manifests as spontaneous vasospasm and can result in brain tissue damage by way of ischemia-reperfusion and development of delayed ischemic neurological deficits [45]. In the case of vasospasm, a further increase in the level of MDA was observed with a slower decrease of neuroprostanes, 4-ONE, and 4-HNE levels compared to patients without vasospasm complications. Lowered systemic levels of ROS and increased antioxidant capacity result from antioxidant treatment strategies following cerebral hemorrhage. This therapy after SAH primarily involves the use of natural antioxidants, including vitamin C and E, melatonin, carotenoids, and micro-minerals as well as drug edaravone [46,47].

## 4. Materials and Methods

### 4.1. Reagents

Drugs and reagents were obtained from the following sources: 8-iso prostaglandin-d_4_ F_2α_-d_4_ (8-isoPGF_2α_–d_4_), 8-iso prostaglandin F_2α_ (8-isoPGF_2α_ - F_2_-isoprostanes), 4-hyroxynonenal (4-HNE), 4-hydroxyhexenal (4-HHE), 4-oxo-nonenal (4-ONE), and malondialdehyde (MDA) from Cayman Chemical Company (Ann Arbor, MI, USA), cis-4,7,10,13,16,19-docosahexaenoic acid (DHA), glutathione peroxidase (GSH-Px), l-glutathione reduced (GSH), (±) α-tocopherol (vitamin E), all trans retinol (vitamin A), and L-ascorbic acid (vitamin C) from Merck KGaA (Sigma–Aldrich, Darmstadt, Germany).

### 4.2. Materials

**Figure 3:**
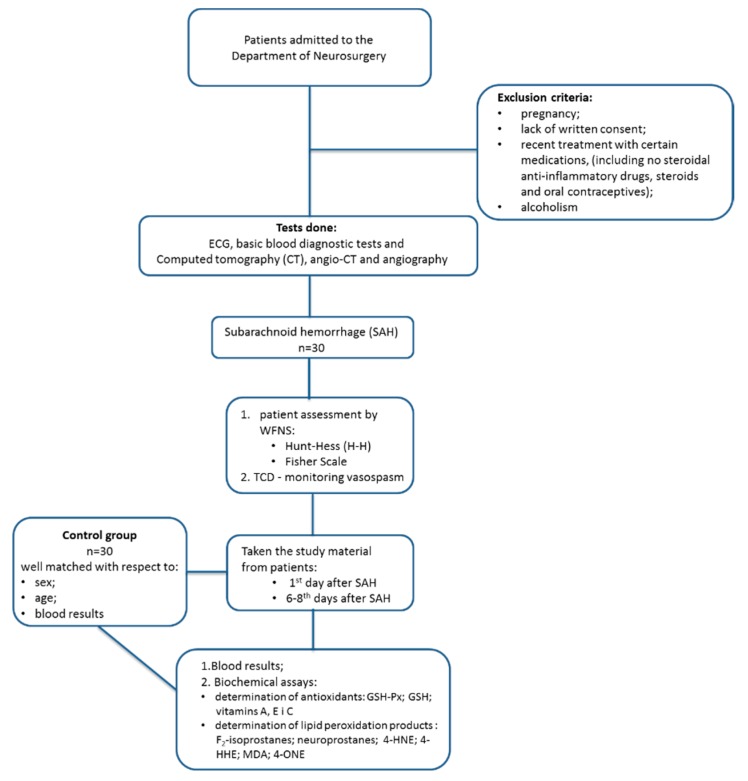
Flowchart of the study.

The blood samples used in this study were collected from a group of 30 patients diagnosed with subarachnoid hemorrhage due to ruptured aneurysm in the Department of Neurosurgery Medical University of Bialystok, Poland, including 21 female with a mean age of 55 (33–67) and 9 men with the mean age of 51 (38–65). The patient recruitment period lasted from December 2014 to September 2017. The hemorrhage was diagnosed with brain CT (computed tomography) and the aneurysm with angio-CT and angiography. The routine tests included ECG and basic blood diagnostic tests. Each SAH-patient during an admission was assessed with the World Federation of Neurological Surgeons Scale (WFNS), the Hunt–Hess scale (H–H), and the Fisher Scale [48,49]. During hospitalization in the course of SAH the vasospasm was monitored with transcranial Doppler (TCD) [50]. The study material was taken from patients after 1 day and then 6 to 8 days after SAH to compare changes in the body over time. The control group consisted of 30 healthy subjects (21 women and 9 men; average age 53 (31–64). The groups were well matched with respect to sex, age, and blood results. Results of the analyzed parameters of healthy subjects and SAH-patients are outlined in Table 3. The exclusion criteria were as follows: pregnancy; lack of written consent; recent treatment with certain medications, including nonsteroidal anti-inflammatory drugs, steroids, and oral contraceptives; and alcoholism (Figure 3).

The study commenced after obtaining approval from the Local Bioethics Committee at the Medical University of Bialystok (Poland) (R-I-002/70/2014), and written informed consent was obtained from all patients.

### 4.3. Samples and Laboratory Measurements

Blood samples were drawn by venipuncture and collected into heparinized and non-heparinized tubes. Samples were centrifuged at 2.000× *g*, 4 °C for 20 min to obtain plasma and serum. An antioxidant—butylhydroxytoluene (BHT)—was added to plasma samples before storing them to prevent oxidation. Samples were stored at −80 °C until analysis.

Serum samples were used to perform laboratory measurements including morphology (Table 3).

### 4.4. Biochemical Assays

#### 4.4.1. Determination of Antioxidants

The GSH-Px (EC.1.11.1.6) activity was assessed spectrophotometrically in the plasma [51]. One unit of GSH-Px activity was defined as the amount of enzyme catalyzing the oxidation of 1 µmol NADPH min^−1^ at 25 °C and pH 7.4.

The HPLC methods were used to determine the level of vitamins A and E and vitamin C [52,53]. The vitamins A and E were extracted from plasma with hexane containing 0.025% butylated hydroxytoluene. The hexane phase was removed and dried with sodium sulfate, and 50 µL of the hexane extract was injected on the column. The analyses were performed on an HPLC system (Agilent, Santa Clara, USA) with a diode array detector (λ = 294 nm) using a RP C18 column. The mobile phase contained 5/95 (*v/v*) water–methanol. To determine ascorbic acid content, plasma was mixed with an equal volume of metaphosphoric acid. Before analysis, samples were centrifuged (1000× *g*, 10 min) to remove precipitated protein after which the samples were immediately assayed. Separation was performed on a RP C18 column and UV detection at 250 nm was applied.

Glutathione was quantified using the capillary electrophoresis (CE) method of Maeso et al. [54]. Samples were sonificated in Eppendorf tubes with 2 mL of a mixture containing ACN/H_2_O (62.5:37.5, *v/v*) and centrifuged at 29,620 g for 10 min. The supernatant was immediately measured with CE. The separation was performed on a capillary with 47 cm total length (40 cm effective length) and 50 m i.d. and was operated at 27 kV with UV detection at 200 ± 10 nm. The GSH concentration was determined using a calibration curve range: 1–120 nmol/L (*r^2^* = 0.9985).

#### 4.4.2. Determination of Lipid Peroxidation Products

The reactive aldehydes (4-HNE, 4-HHE, MDA, 4-ONE) generated during lipid peroxidation were determined using a GC–MS system (7890A GC–7000 with QqQ spectrometer, Agilent Technologies, Palo Alto, CA, USA), and the *O*-pentafluorobenzyl-oxime (*O*-PFB-oxime) or *O*-pentafluorobenzyl-oxime-trimethylsilane (*O*-PFB-oxime-TMS) derivatives, based on Luo’s method with minor modifications [55]. Benzaldehyde-d6 (50 pmol) was used as an internal standard. Derivatized aldehydes were separated using an HP-5ms capillary column with a temperature gradient. All aldehydes were analyzed in selected ion-monitoring (SIM) mode. The LOD for oxime derivatives of aldehydes in the range of 2–8 pmol/mL, and the LOQ was in the range of 4–20 pmol/mL, at a signal-to-noise ratio of 3 and 10, respectively. The precision of LOQ was 7.5% (CV). The linear dynamic ranges were: 0.004–0.100 nmol/mL for 4-HHE, 0.05–20.00 nmol/mL for 4-HNE and 4-ONE, and 0.5–700.0 nmol/mL for MDA.

Total F_2_-isoprostanes (8-isoPGF2α) and neuroprostanes (NPs) were quantified using the modified LC–MS methods of Coolen and Fam respectively [56,57]. In short, F_2_-isoprostanes and neuroprostanes were isolated using the SPE method, after an alkaline hydrolysis step. All analyses were performed using an Agilent 1290 UPLC system interfaced with an Agilent 6460 QqQ spectrometer with electrospray ionization source (ESI). The separation was performed using a reverse phase C18 column and linear gradient with water (pH 5.7) and acetonitrile. F_2_-isoprostanes–d4 (8-isoPGF2α-d4) as an internal standard was used. F_2_-isoprostanes was analyzed in negative-ion mode using MRM (multiple reaction monitoring). In case of free F_2_-isoprostanes quantifying an alkaline hydrolysis step was omitted. The plasma F_2_-isoprostanes were determined as free F_2_-isoprostanes (hydrolyzed in the organism), non-esterified in phospholipids, and as total F_2_-isoprostanes containing free and esterified F_2_-isoprostanes. The LOD was 30 pg/mL, at a signal-to-noise ratio of 3. The LOQ for the standard F_2_-isoprostane was 60 pg/mL, at a signal-to-noise ratio of 10. The precision of LOQ was 8.25% (CV). The linear dynamic range was 60–6000 pg/mL. Neuroprostanes were analyzed by selected ion monitoring (SIM) in the *m/z* 357, as a series of peaks that have molecular masses and retention times expected for neuroprostanes generated from the oxidation of DHA in vitro.

### 4.5. Statistical Analysis

Data obtained in the current study were expressed as mean ± SD. One-way analysis of variance (ANOVA) with Bonferroni post hoc tests were used to determine significant differences between healthy subjects and groups of patients (GraphPad Software, San Diego, CA, USA). Differences were considered significant if *p* < 0.05 (Figure 1 and Figure 2). For comparisons between patients groups, the chi-square test was used for categorical variables. The normal distribution of quantitative data was verified using the Kolmogorov–Smirnov test with corrections performed using the Lilliefors test and the Shapiro–Wilk test. To compare differences between the groups, the Mann–Whitney U test and Kruskal–Wallis test were used. For the comparison of dependent variables, Friedman’s test was used with an adjusted Conover post-hoc test. A *p* value of <0.05 was considered statistically significant. All statistical analyses were performed using Stata/IC 13.0 (StataCorp, College Station, TX, USA) (Table 1 and Table 2).

## 5. Conclusions

Worse clinical and neurological conditions after SAH can result from the presence of cerebral edema, major bleeding, intracranial hematoma, or vasospasm. This corresponds to a reduced antioxidant defense and, consequently, increased lipid peroxidation and slower regression of the observed changes. These observations confirm that biochemical parameters can be a diagnostic and prognostic element supporting clinical prognosis. Given the lack of explicit diagnostic parameters for assessing the condition of patients after SAH, the correlation of biochemical results with the parameters of clinical assessment of patients provide information on the medical consequences of oxidative stress observed after cerebral hemorrhage. It can be concluded that monitoring of neuroprostanes, 4-ONE, and MDA levels may be of particular importance in assessing vasospasm. Indicated lipid peroxidation products may provide support in monitoring the condition of patients 

## Figures and Tables

**Table 1:** The mean plasma concentration of antioxidants in SAH-patients with regard to neurological conditions and gender; ^a^ *p* < 0.05 in comparison with patients on the first day after SAH.

	Parameters	GSH-Px (uU/mg Protein)		GSH (nmol/mL)		Vit. E (ng/mL)		Vit. A (ng/mL)		Vit. C (ng/mL)	
	Day	1st	6–8th	1st	6–8th	1st	6–8th	1st	6–8th	1st	6–8th
**Focal signs**	/-/ *n* = 25	408 ± 119	393 ± 138	10.0 ± 0.9	7.0 ± 0.7 ^a^	313 ± 91	294 ± 101	43.7 ± 13.9	38.0 ± 12.5	23.6 ± 13.4	27.3 ± 11.7
	/+/ *n* = 5	438 ± 168	371 ± 106	10.3 ± 0.9	7.2 ± 0.7 ^a^	364 ± 69	298 ± 94	55.2 ± 18.6	33.1 ± 6.3 ^a^	35.1 ± 17.1	27.3 ± 14.1
**Hunt-Hess scale**	/1/ *n* = 19	415 ± 141	387 ± 98	9.9 ± 0.8	6.8 ± 0.7 ^a^	312 ± 94	279 ± 97	41.8 ± 13.7	37.3 ± 13.1	26.4 ± 10.7	28.0 ± 11.3
	/2-4/ *n* = 11	387 ± 98	390 ± 164	10.2 ± 0.9	7.3 ± 0.5 ^a^	312 ± 92	281 ± 79	48.0 ± 16.7	40.9 ± 10.9	29.2 ± 13.0	27.2 ± 10.5
**WFNS**	/1/ *n* = 23	418 ± 138	402 ± 134	10.1 ± 0.9	7.0 ± 0.7 ^a^	315 ± 94	287 ± 101	42.5 ± 14.3	37.5 ± 12.5	27.4 ± 11.0	28.4 ± 11.5
	/2-4/ *n* = 7	394 ± 102	332 ± 122	9.9 ± 0.8	7.2 ± 0.7 ^a^	284 ± 107	284 ± 82	43.1 ± 21.9	41.5 ± 11.4	31.1 ± 15.8	30.1 ± 12.5
Fisher scale	/1-2/ *n* = 11	407 ± 133	344 ± 98	9.9 ± 0.8	7.3 ± 0.6 ^a^	308 ± 94	279 ± 97	41.4 ± 13.7	37.3 ± 13.1	26.4 ± 10.7	28.0 ± 11.3
	/3-4/ *n* = 19	414 ± 129	407 ± 145	10.3 ± 0.9	6.6 ± 0.7 ^a^	412 ± 165	325 ± 184	53.7 ± 37.9	38.2 ± 17.6	23.8 ± 10.6	25.5 ± 10.7
**Brain edema**	/-/ *n* = 11	391 ± 135	355 ± 81	10.2 ± 0.9	7.2 ± 0.6 ^a^	308 ± 94	279 ± 97	41.4 ± 13.7	37.3 ± 13.1	26.4 ± 10.7	28.0 ± 11.3
	/+/ *n* = 19	424 ± 126	402 ± 153	9.9 ± 0.9	6.7 ± 0.7 ^a^	397 ± 173	349 ± 161	56.0 ± 38.6	38.7 ± 16.3 ^a^	28.9 ± 10.3	27.3 ± 12.0
**Hematoma**	/-/ *n* = 25	400 ± 125	375 ± 121	10.0 ± 0.9	7.0 ± 0.7 ^a^	324 ± 91	290 ± 101	43.7 ± 13.9	37.6 ± 12.5	26.8 ± 10.7	27.3 ± 11.7
	/+/ *n* = 5	473 ± 143	437 ± 187	10.3 ± 0.9	7.2 ± 0.5 ^a^	308 ± 83	275 ± 72	45.5 ± 9.2	44.3 ± 13.8	30.1 ± 10.5	26.1 ± 12.0
**Complications**	/-/ *n* = 25	406 ± 134	376 ± 129	10.1 ± 0.9	7.0 ± 0.7 ^a^	324 ± 91	290 ± 101	43.7 ± 13.9	37.6 ± 12.5	26.8 ± 10.7	27.3 ± 11.7
	/+/ *n* = 5	455 ± 62	430 ± 157	9.9 ± 0.9	7.2 ± 0.6 ^a^	373 ± 150	368 ± 193	40.0 ± 9.2	47.7 ± 16.3	26.8 ± 5.2	24.9 ± 2.9
**Gender**	/W/ *n* = 21	440 ± 132	385 ± 138	9.9 ± 0.8	7.1 ± 0.6 ^a^	355 ± 134	337 ± 113	44.4 ± 14.4	42.0 ± 12.1	29.1 ± 12.9	26.3 ± 10.7
	/M/ *n* = 9	347 ± 96	387 ± 126	10.3 ± 0.9	6.8 ± 0.9 ^a^	327 ± 107	218 ± 103 ^a^	58.6 ± 42.4	32.3 ± 15.1 ^a^	29.4 ± 10.3	28.1 ± 11.4

W-women; M-men.

**Table 2:** The mean plasma concentration of lipid peroxidation products in SAH-patients with regard to neurological conditions and gender; ^a^ *p* < 0.05 in comparison with patients on the first day after SAH.

	Parameters	F_2_-isoprostanes [ng/ml]		Neuroprostanes [ng/ml]		4-HNE [nmol/ml]		4-HHE [pmol/ml]		MDA [nmol/ml]		4-ONE [pmol/ml]	
	Day	1st	6–8th	1st	6–8th	1st	6–8th	Day	1st	6–8th	1st	6–8th	1st
**Focal signs**	/-/ *n* = 25	1.12 ± 0.55	0.86 ± 0.30 ^a^	6.11 ± 2.62	3.88 ± 2.17 ^a^	0.57 ± 0.39	0.28 ± 0.21 ^a^	3.36 ± 1.58	3.65 ± 1.51	7.05 ± 4.98	5.74 ± 3.88	0.19 ± 0.15	0.15 ± 0.12
	/+/ *n* = 5	1.17 ± 0.85	0.71 ± 0.41	7.11 ± 2.65	4.14 ± 1.08 ^a^	0.94 ± 0.62	0.24 ± 0.14 ^a^	2.90 ± 1.03	3.20 ± 1.47	8.90 ± 6.25	6.63 ± 4.82	0.20 ± 0.13	0.13 ± 0.03
**Hunt-Hess scale**	/1/ *n* = 19	1.13 ± 0.49	0.85 ± 0.40 ^a^	6.25 ± 2.82	3.75 ± 1.83 ^a^	0.62 ± 0.45	0.25 ± 0.16 ^a^	3.51 ± 1.87	3.78 ± 1.41	6.22 ± 4.37	5.17 ± 3.07	0.15 ± 0.11	0.14 ± 0.10
	/2-4/ *n* = 11	1.11 ± 0.78	0.84 ± 0.37	6.04 ± 2.22	4.36 ± 2.71	0.69 ± 0.49	0.34 ± 0.26 ^a^	3.05 ±1.30	2.84 ± 1.14	9.04 ± 5.46	7.74 ± 5.17	0.25 ± 0.18	0.17 ± 0.15
**WFNS**	/1/ *n* = 23	1.02 ± 0.50	0.83 ± 0.42	6.02 ± 2.72	4.08 ± 2.26	0.66 ± 0.47	0.29 ± 0.22 ^a^	3.17 ± 1.48	3.42 ± 1.27	6.31 ± 4.19	5.73 ± 3.51	0.19 ± 0.14	0.16 ± 0.11
	/2-4/ *n* = 7	1.32 ± 0.76	0.83 ± 0.32	6.42 ± 2.00	3.36 ± 1.17 ^a^	0.60 ± 0.38	0.25 ± 0.13 ^a^	3.39 ± 1.17	3.87 ± 1.35	9.28 ± 6.59	6.98 ± 5.22	0.19 ± 0.16	0.10 ± 0.09
**Fisher scale**	/1-2/ *n* = 11	1.05 ± 0.57	0.78 ± 0.44	6.36 ± 3.42	4.00 ± 2.12 ^a^	0.66 ± 0.46	0.30 ± 0.20 ^a^	4.11 ± 1.79	4.34 ± 1.99	7.47 ± 4.99	4.64 ± 3.12	0.15 ± 0.12	0.15 ± 0.10
	/3-4/ *n* = 19	1.13 ± 0.61	0.86 ± 0.37	5.99 ± 2.04	3.89 ± 2.12 ^a^	0.64 ± 0.45	0.27 ± 0.21 ^a^	2.85 ± 1.15	3.04 ± 1.21	6.85 ± 5.03	6.82 ± 4.24	0.21 ± 0.15	0.15 ± 0.12
**Brain edema**	/-/ *n* = 11	1.11 ± 0.54	0.89 ± 0.44	5.63 ± 1.65	3.73 ± 1.45 ^a^	0.71 ± 0.53	0.23 ± 0.19 ^a^	4.28 ± 1.64	3.93 ± 1.43	6.88 ± 5.36	5.07 ± 3.73	0.14 ± 0.13	0.16 ± 0.10
	/+/ *n* = 19	1.09 ± 0.62	0.79 ± 0.30 ^a^	6.42 ± 2.98	4.05 ± 2.42 ^a^	0.62 ± 0.42	0.31 ± 0.20 ^a^	2.57 ± 1.35	3.33 ± 1.60	7.13 ± 4.87	6.51 ± 4.09	0.21 ± 0.15	0.15 ± 0.12
**Hematoma**	/-/ *n* = 25	1.09 ± 0.55	0.88 ± 0.40	6.16 ± 2.67	3.73 ± 1.63 ^a^	0.59 ± 0.44	0.26 ± 0.15 ^a^	3.53 ± 1.41	3.75 ± 1.31	6.64 ± 5.02	5.40 ± 3.61	0.16 ± 0.12	0.13 ± 0.09
	/+/ *n* = 5	1.14 ± 0.85	0.66 ± 0.35	5.85 ± 1.97	5.22 ± 4.08	0.92 ± 0.44	0.39 ± 0.35	1.84 ± 0.40	2.38 ± 0.50	8.92 ± 4.51	9.74 ± 4.34	0.30 ± 0.20	0.29 ± 0.18
**Complications**	/-/ *n* = 25	1.16 ± 0.56	0.89 ± 0.37	6.32 ± 2.59	3.80 ± 1.90 ^a^	0.64 ± 0.46	0.24 ± 0.12 ^a^	3.42 ± 1.26	3.74 ± 1.74	7.40 ± 5.24	5.92 ± 3.96	0.18 ± 0.14	0.13 ± 0.09
	/+/ *n* = 5	0.57 ± 0.51	0.33 ± 0.04	5.00 ± 2.22	4.56 ± 2.99	0.71 ± 0.38	0.46 ± 0.38 ^a^	1.32 ± 0.34	2.03 ± 0.56	5.47 ± 3.06	6.70 ± 4.42	0.23 ± 0.14	0.22 ± 0.17
**Gender**	/W/ *n* = 21	0.98 ± 0.54	0.78 ± 0.38	6.36 ± 2.78	3.94 ± 1.90 ^a^	0.67 ± 0.42	0.25 ± 0.16 ^a^	3.01 ± 1.60	3.33 ± 1.63	6.89 ± 4.94	6.17 ± 4.02	0.16 ± 0.13	0.14 ± 0.10
	/M/ *n* = 9	1.38 ± 0.62	0.92 ± 0.42 ^a^	5.30 ± 1.40	3.91 ± 2.67	0.66 ± 0.42	0.35 ± 0.26	3.76 ± 2.19	4.01 ± 2.00	7.53 ± 5.28	5.78 ± 4.13	0.25 ± 0.16	0.18 ± 0.16

W-women; M-men.

**Table 3:** Results of blood laboratory tests of healthy subjects and patients with SAH.

Parameters		Healthy Subjects *n* = 30	SAH Patients *n* = 30
WBC (103/mm^3^)		5.21 ± 1.12	11.15 ± 3.67
RBC (106/µl)		4.21 ± 0.34	4.09 ± 0.52
PLT (×100,000/mm^3^)		167 ± 42	203 ± 61
Sex	male:female	9:21	9:21
Age	<50 y:>50 y	13:17	13:17
Number of aneurysms 1:>1		-	21:9
Size of aneurysm <10 mm:>10 mm		-	26:4
Embolization:Clipping		-	19:11
